# Very heavy dark Skyrmions

**DOI:** 10.1140/epjc/s10052-017-5415-3

**Published:** 2017-12-07

**Authors:** Rainer Dick

**Affiliations:** Department of Physics and Engineering Physics, University of Saskatchewan, 116 Science Place, Saskatoon, SK S7N 5E2 Canada

## Abstract

A dark sector with a solitonic component provides a means to circumvent the problem of generically low annihilation cross sections of very heavy dark matter particles. At the same time, enhanced annihilation cross sections are necessary for indirect detection of very heavy dark matter components beyond 100 TeV. Non-thermally produced dark matter in this mass range could therefore contribute to the cosmic $\gamma$-ray and neutrino flux above 100 TeV, and massive Skyrmions provide an interesting framework for the discussion of these scenarios. Therefore a Higgs portal and a neutrino portal for very heavy Skyrmion dark matter are discussed. The Higgs portal model demonstrates a dark mediator bottleneck, where limitations on particle annihilation cross sections will prevent a signal from the potentially large soliton annihilation cross sections. This problem can be avoided in models where the dark mediator decays. This is illustrated by the neutrino portal for Skyrmion dark matter.

## Introduction

The fact that direct search experiments so far could not confirm a dark matter signal in the theoretically well motivated WIMP mass range between about 10 GeV and a few TeV creates increasing pressure to look for light dark matter particles or for very heavy dark matter as alternative explanations of the dark matter puzzle. Light dark matter models can be motivated through axions, dilatons, or moduli fields in string theory, and they will be tested by upcoming experiments.

On the other hand, superheavy dark matter with masses above $1\,\mathrm {EeV}=10^9\,\mathrm {GeV}$ had been discussed extensively as a consequence of initial lack of observation of a Greisen–Zatsepin–Kuzmin (GZK) cutoff [1, 2] in the ultra-high-energy cosmic ray spectrum [3–6]. The early pioneering paper on ultra-high-energy cosmic rays from superheavy dark matter was Hill’s paper on monopolonium decay [7]. However, the discovery of a GZK cutoff in the meantime [8–11], the successful matching of the spectrum above $3\,\mathrm {EeV}$ in terms of nuclear components [12], and the increasingly stringent limits on the fluxes of ultra-high-energy neutrinos [13] and photons [14, 15], indicate that superheavy dark matter, if it exists, will not be detectable in direct or indirect search experiments as we know them. This is not entirely unexpected, since we knew from the start that the unitarity limits on the annihilation cross section of superheavy dark matter make it an ideal candidate for practically secluded dark matter in terms of particle physics experiments [6].

However, there is a mass range between the WIMP mass range and the superheavy mass range that warrants further exploration. We are particularly interested in the very heavy dark matter mass range between about 100 TeV and $10\,\mathrm {PeV}=10^7\,\mathrm {GeV}$ because of the possibility that there might be a detectable flux of very high-energy $\gamma$-rays between 100 TeV and a few PeV, and because IceCube has seen neutrinos with PeV scale energies [16, 17]. A detectable contribution from dark matter annihilation in this energy range calls for solitonic enhancement of annihilation cross sections, because the indirect search limits in the TeV mass range are already encroaching on the thermal production limit [18]. This makes indirect dark matter signals from dark particle annihilation for higher masses very unlikely, as these signals can be expected to drop with dark matter mass *M* roughly in proportion to $M^{-3}$, i.e. faster than the decrease of the cosmic $\gamma$-ray flux with energy. The indirect search limits in the TeV mass range therefore pose the question whether there is any hope for a potentially detectable indirect dark matter $\gamma$-ray signal in the energy range beyond 100 TeV, which could be motivated by theory. Solitonic states can avoid this negative verdict on indirect signals from very heavy dark matter, because their annihilation cross sections are size limited $\sigma \propto L^2$ rather than mass limited $\sigma \propto M^{-2}$. Non-topological dark solitons could arise as e.g. as dark Q-balls [19–27]. However, in the present paper we focus on Skyrmions as an example of topological dark solitons.

Sommerfeld enhancement provides another way to achieve high cross section values and potentially observable indirect signals for heavy dark matter if the dark matter particles participate in interactions which are long range compared to their Compton wavelength $M^{-1}$ [28]. This applies especially to heavy dark matter which is weakly charged [29–37], or to heavy Majorana dark matter which can exchange scalar bosons of lower mass. However, we will see that the size-induced solitonic enhancement factors can reach levels of order $10^7$ for Skyrmion couplings of order $g_V\gtrsim 0.1$, and therefore the size effect alone will be sufficient to generate observable signals from very heavy dark Skyrmions. Therefore we develop very heavy dark Skyrmion models where the Skyrmions are not charged under long range gauge interactions, although charged very heavy Skyrmions are an interesting topic for further investigations.

The question for indirect signals from very heavy dark matter is a timely question to address, since IceCube is already exploring neutrinos in the PeV energy range while the Cherenkov telescope array (CTA) will start to explore cosmic $\gamma$-rays in the energy range beyond 100 TeV. Searches for indirect dark matter signals will likely remain the primary, if not the only option to explore the mass range beyond 100 TeV in the foreseeable future [38]; see also [39] for an excellent discussion of motivation and mass reach of next generation colliders.

The lower end of the heavy mass range poses interesting theoretical challenges which force us to deviate from standard dark matter theory when we cross this boundary. Indeed, it is well known that standard thermal creation of dark matter particles would imply overclosure of the universe if the dark matter particles are heavier than about 100 TeV [40]. This problem could eventually be ameliorated by dilution of dark matter through late time entropy production [41, 42]. However, there is also another, less well known problem appearing at about 10–50 TeV, depending on the dark matter model: Standard thermal dark matter creation also requires increasingly strong dark matter coupling $g_{\mathrm{D-SM}}\equiv g$ of the dark matter to Standard Model particles to produce heavier particles. Once we reach a mass of a few ten TeV, the required coupling to standard model particles needs to be of non-perturbative strength $g_{\mathrm{D-SM}}\sim 4\pi$ for thermal dark matter creation. The reason for this is that the thermally averaged annihilation cross section at temperature *T* [43],

1 $$\begin{aligned} \nonumber \langle \sigma v\rangle _{G}(M,T)= & {} \int _{4M^2}^\infty \!\mathrm{d}s\,K_1(\sqrt{s}/T)\sqrt{s}\left( s-4M^2\right) \\&\times \frac{\sigma (s)}{8M^4 T K_2^2(M/T)}, \end{aligned}$$

varies asymptotically like $M^{-2}$ for large dark matter mass, whereas the required cross section $\langle \sigma v\rangle _{r}(T_f)$ for thermal dark matter creation at the freeze-out temperature $T_f$ only varies logarithmically with dark matter mass. Every perturbative dark matter model leads to a prediction for $\sigma (s)=g^2\times \sigma (s)|_{g=1}$, and thus to a prediction for $\langle \sigma v\rangle _{G}(M,T_f)=g^2\times \langle \sigma v\rangle _{G}(M,T_f)|_{g=1}$, whereas $\langle \sigma v\rangle _{r}(T_f)$ is determined by cosmology. This leads to the prediction of the mass–coupling relations for dark matter models

$$\begin{aligned} g^2(M)=\langle \sigma v\rangle _{r}(T_f)/\langle \sigma v\rangle _{G}(M,T_f)|_{g=1}, \end{aligned}$$

and the quadratic decrease in $\langle \sigma v\rangle _{G}(M,T_f)|_{g=1}$ with mass leads to a linear increase in the required dark matter coupling *g*(*M*) for thermal dark matter creation of very heavy dark matter. Due to the perturbative approximation $\sigma (s)=g^2\times \sigma (s)|_{g=1}$, this reasoning cannot be extended for calculating the required dark matter couplings in the non-perturbative high-mass regime, but it tells us for each model a mass bound where perturbative dark matter theory breaks down.

Contrary to the breakdown in perturbativity of dark matter models for high dark matter mass, the overclosure problem does not involve any perturbative calculation and only assumes that particle creation and annihilation is described by a scattering matrix [40]. However, both the calculation of *g*(*M*) and the overclosure problem assume thermal creation of dark matter. Therefore both the overclosure problem and the need for non-perturbative coupling can be avoided through non-thermal creation of very heavy dark matter. Non-thermal dark matter creation can be achieved in several scenarios during or immediately after inflation [44, 45]. Gravitational production due to the rapidly evolving scale factor during inflation or near its end is a promising possibility [46–51]. Other mechanisms for very heavy dark matter production include the preheating [52, 53] and the reheating phases [46, 54, 55] at the end of inflation, or resonant production due to an evolving effective mass term from couplings to the inflaton through Yukawa type couplings [56, 57] or kinetic couplings [58]. These are different proposals using different physical models for non-thermal dark matter creation in the early universe. However, there is one common denominator that is worth emphasizing: None of these proposals needs to describe the creation dynamics through a scattering matrix and corresponding reaction cross sections, and the standard thermal freeze-out estimate $\Omega _X\propto \langle \sigma v\rangle ^{-1}$ [59] for the remnant dark matter abundance does not apply. Indeed, all the possible mechanisms for very heavy dark matter production are inherently semi-classical, either through directly integrating coupled systems of Lagrangian evolution equations or by evolving Bogolubov coefficients in a rapidly evolving classical spacetime.

This apparent semi-classical aspect of very heavy dark matter generation matches nicely with the observation that a detectable indirect signal in the cosmic $\gamma$-ray and neutrino flux beyond 100 TeV would favor a solitonic component in very heavy dark matter. To elucidate this point, we note that the spectral flux of cosmic rays from annihilation of heavy particle dark matter of density $n_X(\varvec{r})$ and dark anti-matter of density^1^ $n_{\overline{X}}(\varvec{r})$,

2 $$\begin{aligned} j_X(E)=\frac{\mathrm{d}\mathcal {N}(E,2M)}{\mathrm{d}E} \int \mathrm{d}^3\varvec{r}\, \frac{n_X(\varvec{r})n_{\overline{X}}(\varvec{r})}{4\pi |\varvec{r}-\varvec{r}_\odot |^2} v_{X\overline{X}}\sigma _{X\overline{X}}, \end{aligned}$$

can be estimated to scale with dark matter mass *M* roughly like $M^{-3}$, since the densities scale like $M^{-1}$ and the velocity weighted annihilation cross section for very heavy dark matter (i.e. *M* much larger than the top quark mass) scales according to $v_{X\overline{X}}\sigma _{X\overline{X}}\propto M^{-2}$, while more fragmentation products could be expected in proportion to *M* at lower energies. Equation (15) below provides an explicit example for the asymptotic behavior of the velocity weighted annihilation cross section. Comparing the expected drop in cosmic ray flux from annihilation of dark particles with the fact that the combined spectral flux of cosmic rays in all particles scales like $E^{-2.7}$ for $E\lesssim 3$ PeV, tells us that indirect signals from very heavy particle dark matter would be buried deeply in the cosmic ray flux from astrophysical accelerators. On the other hand, the annihilation signal from solitonic dark matter of size $L_S$ and mass $M_S$ would scale like $L_S^2M_S^{-1}$, and could therefore contribute at a detectable level to the cosmic ray flux above 100 TeV. For Skyrmion dark matter the enhancement of the annihilation cross section is of order $L_S^2M_S^2\simeq 7.7\times 10^3 g_V^{-4}$, where $g_V$ is the Skyrmion coupling. This yields an enhancement of order $10^6$ for a weak scale Skyrmion coupling $g_V\simeq 0.3$. This is relevant for indirect dark matter searches beyond 100 TeV. A Higgs portal Skyrmion model is therefore introduced in Sect. 2.

Skyrmions can arise as a consequence of a first order phase transition, e.g. due to chiral symmetry breaking, and Campbell et al. have shown that this can create the correct Skyrmion abundance for dark matter [60]. In these cases, the Skyrmions are indeed the dominant form of energy at least at early stages after the phase transition. However, we will see that a Higgs portal coupling implies that the $\varvec{w}$ bosons, into which the Skyrmions annihilate, will generically also contribute a sizable particle dark matter component if the $\varvec{w}$ bosons are stable. This reduces the observational significance of the Higgs portal model for Skyrmions. To avoid this problem, we also discuss a model where the bosons $\varvec{w}$ decay into $\nu \nu$ and $\overline{\nu }\overline{\nu }$ pairs. We will address this model, which was analyzed for WIMP scale dark matter coupling to very heavy right-handed neutrinos by Dudas et al. [61], as the $\nu ^2$-portal for dark matter, to avoid confusion with the neutrino(-Higgs) portals proposed in [62, 63] (see also [64]). In the neutrino(-Higgs) portal models, unstable dark fermions $\chi _i$ couple to left-handed fermion doublets $\ell _i$ in the Standard Model through the same couplings as the right-handed neutrinos, $\lambda _{ij}\overline{\ell }_i\cdot \tilde{H}\cdot \chi _j+$h.c., where $\tilde{H}=\underline{\epsilon }\cdot H^*=(H^{0*},-H^{+*})$ is the Higgs doublet in the complex conjugate fundamental representation of the electroweak gauge group SU(2), mapped back into the fundamental representation. These models and their generalizations to higher-dimensional operators attracted a lot of interest in recent years due to their possible relevance for PeV scale neutrinos; see [65–70] and the references therein (see e.g. [71–73] for discussion of the possible astrophysical sources of PeV neutrinos). Here we wish instead to couple scalar bosons $w_i$ to the Standard Model through $\lambda _{ijk}\overline{\nu _{i,R}{}^c}\cdot \nu _{j,R}w_k+$h.c., utilizing the fact that the symmetries of the standard model are compatible with Majorana terms for the right-handed neutrinos. The $\varvec{w}$ particles in this model are not the dark matter but are generated from dark Skyrmion annihilation, and they will decay very fast into $\nu _R\nu _R$ pairs or $\overline{\nu }_R\overline{\nu }_R$ pairs, with the right-handed neutrinos then decaying into left-handed neutrinos and Higgs particles. This model has the virtue that Skyrmion annihilation into the $\varvec{w}$ bosons cannot build up a competing dark matter component.

The Higgs portal Skyrmion model is introduced in Sect. 2 and the $\nu ^2$ neutrino portal is introduced in Sect. 3. Section 4 summarizes our conclusions.

## A Higgs portal model for heavy Skyrmion dark matter

Skyrmions have always been part of the toolsets of nuclear theory and mathematical physics, and they have also become very popular in condensed matter physics. Their emergence in black hole physics [74, 75] and as dark matter candidates [76–78] is a relatively recent development that should not surprise us, given that we must naturally encounter low-energy effective field theories in every field of physics. Motivated by techniques of low-energy hadron physics and the observation that we need enhanced annihilation cross sections to see indirect signals of very massive dark matter, we model the dark matter components as Skyrmion excitations of a $\varvec{w}$-field

3 $$\begin{aligned} \nonumber U(x)= & {} \exp [i\varvec{w}(x)\cdot \varvec{\sigma }/f_w] \\= & {} \cos (|\varvec{w}(x)|/f_w)+i\hat{\varvec{w}}(x)\cdot \varvec{\sigma } \sin (|\varvec{w}(x)|/f_w), \end{aligned}$$

where the $\sigma _i$ are the Pauli matrices of a dark SU(2) isospin symmetry, $w_i(x)$ is a triplet of states with the lowest mass in the very massive dark sector above 10 TeV, and $\hat{\varvec{w}}=\varvec{w}/|\varvec{w}|$ is the corresponding unit vector. We couple the resulting dark Skyrmion model to the standard model through a Higgs portal coupling. This will give rise to a two-stage annihilation process of very massive dark Skyrmions into Standard Model states.

Skyrme had proposed [79, 80], and Witten et al. [81, 82] had demonstrated, in the large *N* limit of $\mathrm{SU}(N)$ gauge theory, that baryonic states can be realized as topological excitations of mesonic states, and this reasoning would also apply to a dark gauge theory sector. We will show that this observation is particularly relevant for very heavy dark matter, since it provides a means to enhance the reaction cross sections for very heavy dark matter to observable levels. However, we remain agnostic with respect to the question whether heavy Skyrmion dark matter indeed arises as an effective description of very heavy bound states in a dark gauge theory sector, or as a genuine solitonic excitation of a heavy scalar field. The point is that either way, the resulting enhancement of annihilation cross sections makes this an interesting target for indirect dark matter searches with masses exceeding 100 TeV.

The proposed Skyrme model for heavy dark matter states has a Lagrangian

4 $$\begin{aligned} \mathcal {L}=\mathcal {L}_S+\mathcal {L}_{\mathrm{D-SM}}, \end{aligned}$$

with a Skyrme part

5 $$\begin{aligned} \nonumber \mathcal {L}_S= & {} \frac{1}{16g_V^2}\mathrm {Tr}\left[ \partial ^\mu U\!\cdot \partial ^\nu U^+\!\cdot (\partial _\mu U\!\cdot \partial _\nu U^+-\partial _\nu U\!\cdot \partial _\mu U^+)\right] \\&-\,\frac{f_w^2}{4}\mathrm {Tr}(\partial _\mu U\!\cdot \partial ^\mu U^+) +\frac{m_w^2f_w^2}{2}\mathrm {Tr}(U-1_2), \end{aligned}$$

where the notation $g_V$ for the Skyrme term coupling reminds us that this coupling may arise from vector dominance due to a hidden gauge invariance [83–86] (see also [87]).

The term $\mathcal {L}_{\mathrm{D-SM}}$ describes the coupling to the standard model. A natural guess is to implement Higgs mediation into standard model states. Higgs exchange had been suggested on several occasions as a minimal coupling mechanism between dark matter and the Standard Model [88–92], and a Higgs portal coupling for a very heavy dark Skyrmion model would take the form

6 $$\begin{aligned} \mathcal {L}^{(h)}_{\mathrm{D-SM}}=\frac{g_{wh}f_w^2}{2}(2H^+H-v_h^2)\mathrm {Tr}(U-1_2). \end{aligned}$$

Here *H* is the electroweak Higgs doublet with vacuum expectation value $\langle H^+H\rangle =v_h^2/2$. The coupling (6) would not contribute to any invisible Higgs decay width since all the masses of the dark sector states, including the mass $m_w$ of the $w_i(x)$ fields, are assumed to be much larger than the Higgs mass. It is also not constrained by direct search experiments which are not sensitive to the mass range above 10 TeV. The $\varvec{w}$-Higgs coupling in unitary gauge is

7 $$\begin{aligned} \mathcal {L}^{(h)}_{\mathrm{D-SM}}=g_{wh}f_w^2 v_h\left( h+\frac{h^2}{2v_h}\right) \mathrm {Tr}(U-1_2). \end{aligned}$$

This model differs from little Higgs Skyrmion dark matter models [76, 77] by not assuming that the Higgs field itself is part of the fields $w_i$, which parametrize a coset in the little Higgs models. The model is also different and much less constrained than the model in Ref. [78], where the Higgs field was coupled to the kinetic term $\mathrm {Tr}(\partial _\mu U\cdot \partial ^\mu U^+)$ and the non-linear fields *U*(*x*) were related to the electroweak gauge bosons. These models therefore also did not include the Higgs portal coupling (7).

The scale $f_w$ is a mass scale, but contrary to the hadronic Skyrme models, it is not a decay constant. Recall that the $\pi$ mesons of hadronic physics can only decay because their constituents couple to the lighter lepton sector through the electroweak gauge bosons. There is no corresponding low mass dark matter sector included in the $\varvec{w}$+Skyrmion picture of very heavy dark matter, and the $\varvec{w}$-particles in the model (4, 5) with the coupling (7) are stable up to annihilation into Standard Model states through the Higgs portal. Therefore the dark sector in this type of dark Skyrmion model will generically consist both of $\varvec{w}$ particles and of their Skyrmion excitations. Skyrmion annihilation and thermal creation can generate $\varvec{w}$-particles which will also contribute to the dark matter if they do not annihilate sufficiently fast into Standard Model states. Nevertheless, the Skyrmions can initially be produced as the dominant dark matter component if they arise as a consequence of symmetry breaking during a first order phase transition [60], and this is the assumption used here. However, it is also intriguing to ask what happens if the $\varvec{w}$ particles are not just a low-energy effective description of a condensate in the low-energy effective theory of a broken symmetry, but are thermally produced in the early universe before Skyrmions are generated in a phase transition. For the reasons alluded to above, we can perform a perturbative analysis of this question only if the mass $m_w$ of the $\varvec{w}$ particles is not too large, and we will return to this question once we have assembled the pertinent cross sections.

The Hamiltonian for the Lagrange density (4) is

8 $$\begin{aligned} \mathcal {H}= & {} \frac{1}{16g_V^2}\mathrm {Tr}\left[ \partial _\mu U\!\cdot \partial _\nu U^+\!\cdot \left( \partial _\nu U\!\cdot \partial _\mu U^+-\partial _\mu U\!\cdot \partial _\nu U^+\right) \right] \nonumber \\&+\frac{f_w^2}{4}\mathrm {Tr}\left( \partial _\mu U\!\cdot \partial _\mu U^+\right) -\frac{m_w^2f_w^2}{2}\mathrm {Tr}(U-1_2)-\mathcal {L}_{\mathrm{D-SM}},\nonumber \\ \end{aligned}$$

where the summation over lower pairs of 4-indices is Euclidean, e.g. $\partial _\mu U\!\cdot \partial _\mu U^+\equiv \partial _0 U\!\cdot \partial _0 U^+ +\varvec{\nabla }U\!\cdot \varvec{\nabla }U^+$.

Skyrme had demonstrated that the Lagrangian $\mathcal {L}_S$ generates solitons which are stable due to the topologically conserved current (with the convention $\epsilon ^{0123}=-1$)

9 $$\begin{aligned} W^\mu =\frac{1}{24\pi ^2}\epsilon ^{\mu \nu \rho \sigma }\mathrm {Tr}\left( \partial _\nu U\!\cdot U^+\!\cdot \partial _\rho U\!\cdot U^+\!\cdot \partial _\sigma U\!\cdot U^+\right) , \end{aligned}$$

which he had proposed to use as a baryon current. Of course, in our model $W^\mu$ appears as a Skyrmion current in the very heavy dark sector. The winding number $W=\int \mathrm{d}^3\varvec{x}W^0$ measures how often the mapping *U*(*x*) in Eq. (3) (with the compactifying boundary condition $\lim _{|\varvec{x}|\rightarrow \infty }U(x)=1$) wraps compactified $\mathbb {R}^3$ around the *SU*(2) group manifold $S^3$. The mass and the length scale of $|W|=1$ Skyrmions are $M_S=73 f_w/g_V$ and $L_S=1.2/(g_V f_w)$ [87, 93].

The solitonic nature of Skyrmions implies that they are not described as particle states in the Fock space of the theory, and Skyrmion–Skyrmion interactions are not described by the usual scattering matrix formalism. Instead, their interactions have to be studied through mathematical analysis and numerical integration of the equations of motion, and study of the evolution of the topological density $W^0$ [94–98]. For the annihilation of a Skyrmion *S* and an anti-Skyrmions $\overline{S}$, this leads in particular to the interesting result of sudden onset of annihilation through emission of a few $\varvec{w}$ quanta once the distance between the Skyrmion and the anti-Skyrmion is down to the size of a Skyrmion [94, 95]. It is a classical soliton–soliton interaction effect and determined by soliton size, whence the $\ell$-wave unitarity limit $\sigma _\ell \le 4\pi (2\ell +1)/k^2$ on reaction cross sections from scattering matrices [40] does not apply. On the other hand, the underlying $\varvec{w}$ particles (into which the Skyrmions decay upon annihilation in our adoption of the Skyrmion picture for very heavy dark matter), are the basic quantum excitations of the Hamiltonian (8). Annihilation of very heavy dark matter therefore proceeds in two stages. Size-limited annihilation of the heavy Skyrmions into $\varvec{w}$ particles proceeds into quantum mechanical $\varvec{w}\overline{\varvec{w}}$ annihilation into Standard Model matter through the Higgs portal coupling (6).

The $\varvec{w}$ particles as the basic field quanta in Eq. (4) can annihilate into Higgs bosons, fermions, and gauge bosons through the coupling $\mathcal {L}^{(h)}_{\mathrm{S-DM}}$. The corresponding annihilation cross sections with center of mass energy $\sqrt{s}$ are

10 $$\begin{aligned} \sigma _{ww\rightarrow hh}(s)= & {} \frac{g_{wh}^2\sqrt{s-4m_h^2}}{ 8\pi s\sqrt{s-4m_w^2}} \frac{(s+2m_h^2)^2}{(s-m_h^2)^2+m_h^2\Gamma _h^2}, \end{aligned}$$

11 $$\begin{aligned} \sigma _{ww\rightarrow f\overline{f}}(s)= & {} N_cg_{wh}^2\frac{(s-4m_f^2)^{3/2}}{ 2\pi s\sqrt{s-4m_w^2}}\nonumber \\&\times \frac{m_f^2}{(s-m_h^2)^2+m_h^2\Gamma _h^2}, \end{aligned}$$

with $N_c=1$ for leptons and $N_c=3$ for quarks, and

12 $$\begin{aligned} \sigma _{ww\rightarrow ZZ,W^+W^-}(s)= & {} \frac{g_{wh}^2\sqrt{s-4m_{W,Z}^2} }{4\pi s\sqrt{s-4m_w^2}(1+\delta _z)}\nonumber \\&\times \frac{(s-2m_{W,Z}^2)^2+8m_{W,Z}^4}{ (s-m_h^2)^2+m_h^2\Gamma _h^2}. \end{aligned}$$

Here $\delta _z=1$ for annihilation into *Z* bosons and $\delta _z=0$ for annihilation into $W^+ W^-$. The velocity weighted cross sections are $v\sigma =2\sqrt{1-(4m_w^2/s)}\sigma (s)$ and the thermally averaged annihilation cross section at temperature *T* can be calculated using Eq. (1).

The asymptotic cross sections for $\sqrt{s}$ much larger than the standard model masses are (with $\delta _W=1$ for annihilation into $W^+W^-$ and $\delta _W=0$ otherwise)

13 $$\begin{aligned} v_{ww}\sigma _{ww\rightarrow hh,ZZ,W^+W^-}=\frac{g_{wh}^2}{4\pi s}(1+\delta _W) \end{aligned}$$

and

14 $$\begin{aligned} v_{ww}\sigma _{ww\rightarrow f\overline{f}}=N_c\frac{g_{wh}^2m_f^2}{\pi s^2}, \end{aligned}$$

and therefore the net leading order total cross section into standard model states is

15 $$\begin{aligned} v_{ww}\sigma _{ww}=\frac{g_{wh}^2}{\pi s}. \end{aligned}$$

**Fig. 1:**
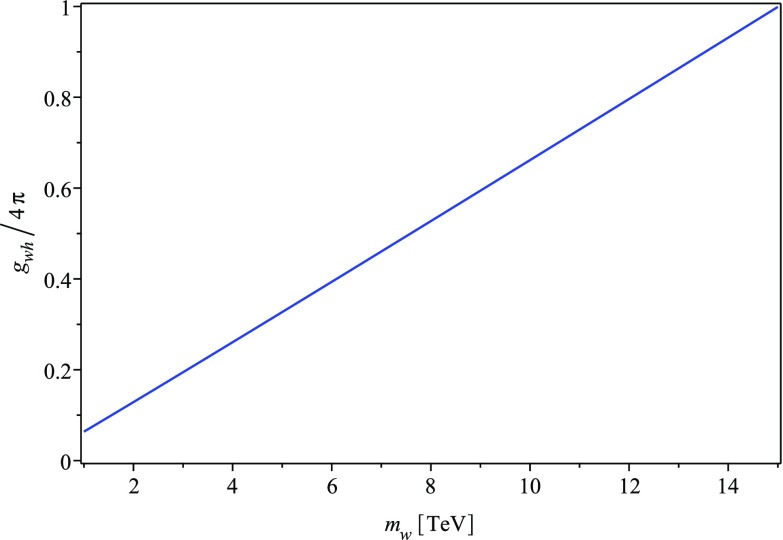
The required Higgs portal coupling for thermal creation of elementary $\varvec{w}$ particles for $1\,\mathrm {TeV}\le m_w\le 15\,\mathrm {TeV}$, if the $\varvec{w}$ particles are the dark matter

With Eq. (15) at hand, we can ask for which very high masses $m\gg m_{\mathrm{top}}$ and couplings $g_{wh}$ the $\varvec{w}$ particles could account for the dark matter in the universe in a scenario of perturbative thermal creation. The requirement of equality of the thermally averaged cross section (1) with the necessary cross section $\langle \sigma v\rangle _{r}(T_f)$ for thermal particle creation yields the relation between $g_{wh}$ and $m_w$ shown in Fig. 1. The $\varvec{w}$ particles could still be thermally created for even higher masses below the unitarity constraint, $m_w\lesssim 100$ TeV, but this would require non-perturbative coupling and could not be analyzed as easily any more. Non-thermal creation could be possible for even higher mass. However, in all these cases, the $\varvec{w}$ particles would be assumed to be the dominant dark matter component with the Skyrmion solutions to (4) as excited states, and the standard unitarity limits on annihilation cross sections of elementary particles would rule out indirect dark matter signals.

Therefore, we will rely in the following on the assumption of Skyrmion production due to a first order phase transition, whence the Skyrmions dominate the energy density at least early after the phase transition [60], and the $\varvec{w}$ fields only parametrize the condensate giving rise to the Skyrmions. In this case the $\varvec{w}$ particles arise from Skyrmion annihilation, and it is a more intriguing question whether the Higgs portal Skyrmion model could provide an indirect dark matter signal. In scenarios of primary creation of the Skyrmions, the effective balance equation for the total $\varvec{w}$ density $n_w=\sum _{i=1}^3 n_{w_i}$ is

16 $$\begin{aligned} \frac{\mathrm{d}n_w}{\mathrm{d}t}= & {} \langle N_w\rangle n_S n_{\overline{S}} v_{S\overline{S}}\sigma _{S\overline{S}} -\frac{1}{6}n_{w}^2 v_{ww}\sigma _{ww}\nonumber \\= & {} \langle N_w\rangle n_S n_{\overline{S}} v_{S\overline{S}}\pi L_S^2 -n_{w}^2\frac{g_{wh}^2}{6\pi \langle s\rangle }, \end{aligned}$$

because the Skyrmion annihilation term will be much larger than the thermal $\varvec{w}$ production term. We use the classical cross section $\sigma _{S\overline{S}}=\pi L_S^2$ for the Skyrmions, noting that the solitons are classical objects of size $L_S$ and using the observation that Skyrmions annihilate when their separation compares to their size [94, 95]. We also note that they typically annihilate into a small number $2\lesssim \langle N_w\rangle \lesssim 7$ of $\varvec{w}$ particles [94, 95], where it is assumed that the mass ratio $M_S/m_w$ is larger than 2. This theoretical result is in agreement with observations of low-energy $p\overline{p}$ annihilations. These annihilations produce $5\pm 1$ pions, with pion multiplicities $3\le n_\pi \le 7$ in 99% of measurements [99]. Skyrmion annihilation will therefore inject relativistic $\varvec{w}$ particles with energies $M_S\ge E_w\ge m_w$ into the ambient $\varvec{w}$-particle density, and $\varvec{w}$ particles will annihilate through the Higgs portal with an average center of mass energy squared $\langle s\rangle$, which will satisfy $4m_w^2\le \langle s\rangle \le 4M_S^2$.

Detailed balance in the equilibrium of very heavy dark matter states then determines the $\varvec{w}$ density,

17 $$\begin{aligned} n_{w}^2= & {} \langle N_w\rangle n_S n_{\overline{S}}\frac{6\pi ^2}{g_{wh}^2} v_{S\overline{S}}L_S^2\langle s\rangle \nonumber \\\ge & {} \langle N_w\rangle n_S n_{\overline{S}}\frac{24\pi ^2}{g_{wh}^2} v_{S\overline{S}}L_S^2 m_w^2. \end{aligned}$$

The expected final products in this scenario will be predominantly photons, light leptons and neutrinos from meson decays, because the primary products from heavy Skyrmion annihilation will be Higgs particles and massive electroweak gauge bosons. The Higgs particles at high energies also predominantly decay into *ZZ* and $W^+ W^-$, and the massive gauge bosons predominantly decay into hadrons, with a dominant meson component which will decay into photons, light leptons and neutrinos. The results of Ref. [100], although derived for lower annihilation energy 10 TeV, are generic for shower composition with annihilation energies well above the top quark mass. The reason is that cross sections into standard model final states simply scale with the relativistic boost factor $\gamma$ like $\sigma _{\mathrm{fi}}\sim 4/s=E^{-2}=\gamma ^{-2}M^{-2}$ once *E* is well above the top quark mass $m_{\mathrm{top}}$. This is a consequence of $v\mathrm{d}\sigma _{\mathrm{fi}}=V\mathrm{d}N_{\mathrm{fi}}/T$ for transitions from an initial 2-particle state $|i\rangle$ into a final $n_f$-particle state $|f\rangle$ with densities of collision partners $V^{-1}$ and reaction rate $\mathrm{d}N_{\mathrm{fi}}/T$. Once $E\gg m_{\mathrm{top}}$ and no more additional Standard Model channels can open up, the Lorentz factors $V\rightarrow V/\gamma$ and $T\rightarrow \gamma T$ determine the scaling with energy, since $v\rightarrow 1$ in a fixed target frame and $v\rightarrow 2$ in the center of mass frame. The follow-up single particle decays scale like $d\Gamma _{\mathrm{fi}}=\mathrm{d}N_{\mathrm{fi}}/T\sim 1/\gamma$, and therefore dominate the shower formation after the initial annihilation channels of the dark matter particles. Therefore the branching ratios of the initial annihilation events and the subsequent decays are practically fixed once $E\gg m_{\mathrm{top}}$.

The CTA is expected to reach a maximum sensitivity $v\sigma \simeq 10^{-26}\,\mathrm {cm}^3/\mathrm {s}$ after 500 h for a dark matter mass at the few TeV scale annihilating to $W^+ W^-$ [38], and then the sensitivity limit scales with mass approximately like $M^3$ for very high dark matter masses. This yields a 500 h sensitivity limit of order $v\sigma \simeq 10^{-25}\mathrm {cm}^3\mathrm {s}^{-1}\times (m/100\,\mathrm {TeV})^3$ at the 100 TeV mass scale and beyond, whereas the Skyrmion value at that scale is

18 $$\begin{aligned} v\sigma _{S\overline{S}\rightarrow W^+ W^-}= & {} 5.8\times 10^{-25}\,\mathrm {cm}^3/\mathrm {s} \times \left( \frac{0.3}{g_V}\right) ^4 \nonumber \\&\times \frac{v}{100\,\mathrm {km}/\mathrm {s}} \times \left( \frac{M_S}{100\,\mathrm {TeV}}\right) ^{-2}. \end{aligned}$$

**Fig. 2:**
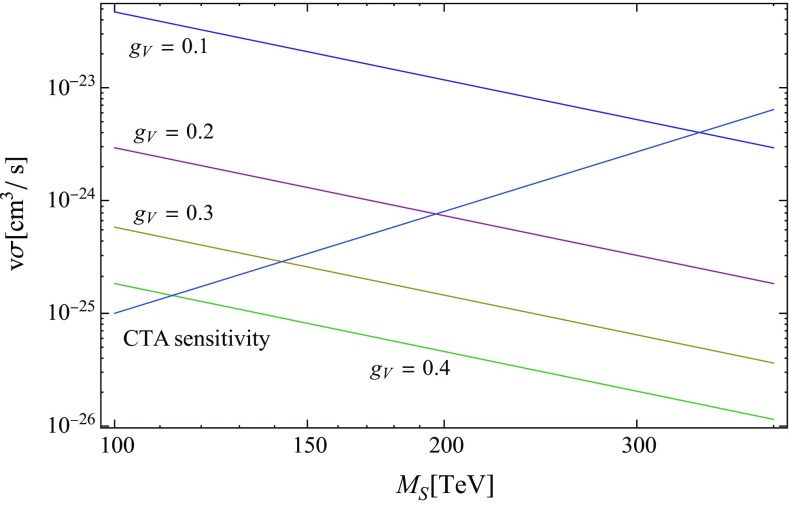
Massive Skyrmion annihilation cross sections to $W^+W^-$ for weak scale couplings compared to the anticipated 500h CTA sensitivity in the mass range $100\,\mathrm {TeV}\le M_S\le 400\,\mathrm {TeV}$ and for $v=100\,\mathrm {km}/\mathrm {s}$

The comparison of very massive Skyrmion dark matter annihilation cross sections with the anticipated CTA sensitivity is displayed in Fig. 2.

Solitonic enhancement factors like the Skyrmion factor $L_S^2M_S^2\simeq 7.7\times 10^3 g_V^{-4}$ are certainly needed for dark matter detection at those mass scales.

However, the Higgs portal to dark Skyrmions demonstrates a dark mediator bottleneck. In terms of mass ratios, the lower bound on $n_w$ in Eq. (17) becomes

19 $$\begin{aligned} n_{w}^2 \ge 2.1\times 10^3\langle N_w\rangle n_S n_{\overline{S}}\frac{\pi ^2}{g_{wh}^2 g_V^2} v_{S\overline{S}}\frac{m_w^2}{M_S^2}. \end{aligned}$$

For weak scale couplings and not excessively small values of $v_{S\overline{S}}$ and $m_w/M_S$, this implies that $n_{w}^2$ should at least be comparable to $n_S n_{\overline{S}}$, such that the Skyrmions would not be the only dark matter component. The Skyrmion annihilation cross sections in Fig. 2 would then have to be reduced by a factor $4n_S n_{\overline{S}}/[(2n_S+n_w)(2n_{\overline{S}}+n_w)]$ in comparison to the anticipated CTA sensitivity. This should likely be a generic feature for soliton enhanced dark matter models. If the carrier field $\varvec{w}$ is an inherently stable quantum field, we should always anticipate that $\varvec{w}$ particles form a comparable or even dominant dark matter component for generic mass ratios and coupling constants, even when the solitonic states are the primary states to be created during a phase transition.

One might hope to avoid this dark mediator bottleneck for indirect signals from dark solitons with stable carrier fields $w_i(x)$ through Sommerfeld enhancement of the annihilation of the carrier fields. The hope would be that this might prevent build-up of a competing particle dark matter component, and enhance the soliton annihilation signal through fast follow-up annihilation of the produced $\varvec{w}$ particles. However, this will not remove the bottleneck because of the hard unitarity limit $\sigma _\ell \le 4\pi (2\ell +1)/k^2$ on the $\ell$-wave annihilation cross sections of particle dark matter of momentum *k*. This implies the lower limit

20 $$\begin{aligned} \frac{n_{w}^2}{n_S n_{\overline{S}}} \ge 1.2\times 10^4\langle N_w\rangle v_{S\overline{S}}v_{ww}\frac{m_w^2}{g_V^4 M_S^2} \end{aligned}$$

on the density ratios, and the required small Skyrmion coupling for an observable indirect signal will always imply a noticeable competing particle dark matter component even for small mass ratio $m_w/M_S$. Suppression of the very high-energy signal from annihilation of very massive dark Skyrmions due to a competing particle dark matter component appears at least generic, if not even inevitable, in models with stable carriers $\varvec{w}$.

## The neutrino portal to Skyrmion dark matter

For $10^4\langle N_w\rangle v_{S\overline{S}}v_{ww}m_w^2/(g_V^4 M_S^2)\gg 1$, the Skyrmions will be a subdominant heavy dark matter component of little observational interest, while at the same time the annihilation cross section for very heavy $\varvec{w}$ particles will be too small to be observable.

A very heavy Skyrmion model with unstable carrier fields $w_i(x)$ avoids this dark mediator bottleneck. Unstable $\varvec{w}(x)$ requires breaking of the vector SU(2) in Eq. (4), but introducing a standard model coupling term proportional to $\varvec{a}\cdot \varvec{w}(x)$ with fixed dark isospin vector $\varvec{a}$ will not do the trick. That would destabilize only the component $w_{\varvec{a}}(x)=\hat{\varvec{a}}\cdot \varvec{w}(x)$, but leave the perpendicular *w* fields stable, such that again an undetectable *w* component could dominate the dark matter sector. Stated differently, the dark vector SU(2) has to be broken completely if we wish to avoid any possible remnant dark $\varvec{w}$ component. A natural way to implement such a scenario of unstable carrier field $\varvec{w}$ for the Skyrmions is to use the broken flavor symmetry in the lepton sector through a Majorana type coupling to the right-handed neutrinos,

21 $$\begin{aligned} \mathcal {L}^{(\nu )}_{\mathrm{D-SM}}= & {} -\,\lambda _{ijk}\overline{\nu ^c_{i,R}}(x)\nu _{j,R}(x)w_k(x) \nonumber \\&-\,\lambda ^*_{ijk}\overline{\nu _{j,R}}(x)\nu ^c_{i,R}(x)w_k(x), \end{aligned}$$

where

$$\begin{aligned} \nu ^c_{i,R}\equiv (\nu _{i,R})^c=\mathcal {C}\cdot \nu ^*_{i,R} \end{aligned}$$

is the charge conjugate of the right-handed neutrino, and we use $\mathcal {C}=i\gamma _2$ for the charge conjugation matrix in the Dirac or Weyl representations of the $\gamma$ matrices. In keeping with the assumption of a very heavy dark sector, we also add a Majorana mass matrix

22 $$\begin{aligned} \mathcal {L}^{(\nu )}_R=-\,\frac{1}{2}M_{R,ij}\overline{\nu ^c_{i,R}}(x)\nu _{j,R}(x) \end{aligned}$$

with large eigenvalues, in addition to the standard Yukawa coupling for mass generation for the isospin 1/2 components of the left-handed lepton doublets $\ell _i=(\nu _{L,i},e^-_{L,i})$,

23 $$\begin{aligned} \mathcal {L}^{(\nu )}_{LR}=-\,\frac{\sqrt{2}}{v_h}m_{D,ij}\overline{\ell }_{i}(x)\cdot \tilde{H}(x)\nu _{j,R}(x)+h.c. \end{aligned}$$

Here $H=(H^+,H^0)$ is the Higgs doublet with $\tilde{H}=(H^{0*},-H^{+*})$, and $v_h=246$ GeV is the Higgs vacuum expectation value.

This yields according to the standard seesaw mechanism mass eigenstates which are mostly right-handed in the high-mass sector and left-handed in the low mass sector, and accounts for the fact that the right-handed neutrinos do not contradict the Planck limit on low mass neutrinos [101]; see e.g. [61, 67]. The heavy right-handed neutrinos will remain in thermal equilibrium for temperatures above a few percent of their masses [102, 103], and could contribute to dark matter if they are stable, or if the eigenvalues of the mixing matrix $m_D\sim \sqrt{m_{\nu }M_R}$ are extremely small to ensure a long lifetime of the heavy right-handed neutrinos, $m_D\lesssim 10^{-14}$ eV. However, for generic eigenvalues of $m_D$ the right-handed neutrinos will decay fast into left-handed neutrinos and Higgs particles, and the left-handed neutrinos could then be seen by the neutrino detectors.

The coupling (21) respects the gauge symmetries and Lorentz invariance of the Standard Model, but we cannot rotate the local dark isospin vector

$$\begin{aligned} \lambda _k(x)=\lambda _{ijk}\overline{\nu ^c_{i,R}}(x)\nu _{j,R}(x) +\lambda ^*_{ijk}\overline{\nu _{j,R}}(x)\nu ^c_{i,R}(x) \end{aligned}$$

with a global vector SU(2) rotation of the $w_i(x)$ fields to make only a particular component decay. The coupling does violate lepton number conservation, but it does not require the right-handed neutrinos to be Majorana neutrinos. It only uses the fact that the local symmetries of the standard model are compatible with a Majorana mass matrix for the right-handed neutrinos. In that sense the $w_i(x)$ could be thought of as dynamical Majorana masses for the right-handed neutrinos, which could generate such mass terms through a Higgs mechanism. Note also that we can choose the Yukawa couplings symmetric in the neutrino indices, $\lambda _{ijk}=\lambda _{jik}$, since the Dirac structure of the vertices is antisymmetric,

$$\begin{aligned} \mathcal {M}=\frac{1+\gamma _5}{2}i\gamma _2\gamma _0=-\mathcal {M}^T. \end{aligned}$$

The Yukawa couplings can therefore be diagonalized with respect to the neutrino flavors or mass eigenstates, if we wish to do so. It makes sense to refer to the coupling (21) as the $\nu ^2$ portal to dark matter, which complements the neutrino(-Higgs) portal introduced in [62, 63].

The coupling (21) yields with $m_w\gg m_{\nu _i}$ averaged $\varvec{w}$-decay rates

24 $$\begin{aligned} \Gamma _{w\rightarrow \nu \nu }=\Gamma _{w\rightarrow \overline{\nu }\overline{\nu }} =\frac{\langle N_w\rangle m_w^2}{96\pi M_S}\sum _{ijk}\left| \lambda _{ijk}\right| ^2, \end{aligned}$$

i.e.

$$\begin{aligned} \Gamma _{w}=\frac{\langle N_w\rangle m_w^2}{48\pi M_S}\sum _{ijk}\left| \lambda _{ijk}\right| ^2, \end{aligned}$$

or an averaged lifetime

25 $$\begin{aligned} \tau _w= & {} 5\times 10^{-28}\,\mathrm {s}\times \left( \sum _{ijk}\left| \lambda _{ijk}\right| ^2\right) ^{-1} \nonumber \\&\times \frac{100\,\mathrm {TeV}}{m_w}\times \frac{2M_S}{\langle N_w\rangle m_w}. \end{aligned}$$

The decay rates and lifetime contain the boost factor $\gamma =2M_S/\langle N_w\rangle m_w$ because the $\varvec{w}$ particles are created with energy $2M_S/\langle N_w\rangle$ from Skyrmion annihilation. The lifetime is very small even for very small Yukawa couplings and large boost factors. There will not be a build-up of a dark $\varvec{w}$ component from Skyrmion annihilation in the neutrino portal model, just as there will not be a build-up of heavy right-handed neutrinos for generic mixing matrix $\underline{m}_D$, and the dark Skyrmions will be the only effective dark matter component in this model.

The approximately monochromatic high-energy neutrinos from the two-step process

$$\begin{aligned} S\overline{S}\rightarrow \langle N_w\rangle \varvec{w} \rightarrow \langle N_w\rangle \nu \nu (\overline{\nu }\overline{\nu }) \end{aligned}$$

will further decay into left-handed neutrinos and Higgs particles, with a coupling strength relative to the right-handed neutrino production of order

$$\begin{aligned} \left( \sum _{ijkl}\left| \lambda _{ijk}m_{D,jl}/v_h\right| ^2\right) \left( \sum _{ijk}\left| \lambda _{ijk}\right| ^2\right) ^{-1}\lesssim 10^{-8}, \end{aligned}$$

because $m_D\sim \sqrt{m_\nu M_R}\lesssim 10$ MeV for neutrino masses $m_\nu <1$ eV [104] and $M_R\lesssim 10$ TeV. This then corresponds to a lifetime estimate $\tau _R\lesssim 5\times 10^{-20}$ s for the heavy right-handed neutrinos. The decay of the right-handed neutrinos is slow compared to the decay of the $\varvec{w}$-particles, but both components are extremely short-lived compared to the stable Skyrmions, which can only decrease in abundance due to annihilation.

Since every high-energy right-handed neutrino decays into a Higgs particle and a high-energy left-handed neutrino, we can estimate the resulting integrated flux of very high-energy neutrinos from Skyrmion annihilation,

26 $$\begin{aligned} \mathcal {F} =\langle N_w\rangle v_{S\overline{S}}\frac{7.7\times 10^3}{g_V^4 M_S^2} \int \mathrm{d}^3\varvec{r}\, \frac{n_X(\varvec{r})n_{\overline{X}}(\varvec{r})}{2|\varvec{r}-\varvec{r}_\odot |^2}. \end{aligned}$$

**Fig. 3:**
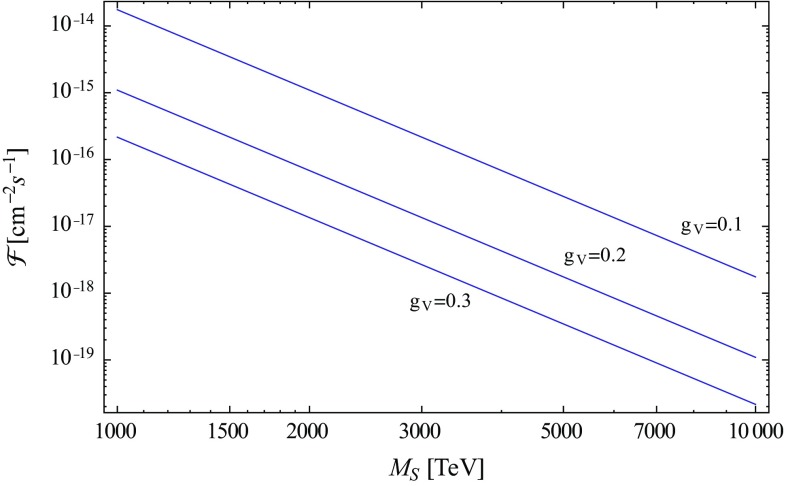
Integrated very high-energy neutrino fluxes from Skyrmion annihilation as a function of Skyrmion mass and for weak scale couplings. The calculation assumes $\langle N_w\rangle =4$, $v=100\,\,\mathrm {km}/\mathrm {s}$, and an NFW halo with $r_s=10.7$ kpc and mass $7.3\times 10^{11}\,M_{\odot }$; see text

Evaluation for a Navarro–Frenk–White (NFW) halo [105, 106],

27 $$\begin{aligned} n_S(\varvec{r})=n_{\overline{S}}(\varvec{r})=\frac{4n_S(r_s)r_s^3}{r(r+r_s)^2} \end{aligned}$$

with $r_\odot =8$ kpc, a scale radius $r_s=10.7$ kpc and a halo mass $M_h=7.3\times 10^{11}M_\odot$ within 385 kpc for the Milky Way [107] yields the fluxes reported in Fig. 3.

The overall diffuse neutrino flux between 250 TeV and 2.5 PeV can be estimated from the IceCube spectral neutrino flux per steradian [16] as $j_\nu \simeq 3.3\times 10^{-13}\,\mathrm {cm}^{-2}\mathrm {s}^{-1}$. The model therefore appears to be compatible with IceCube observations and the assumption that the very high-energy neutrino flux is dominated by astrophysical sources.

## Conclusions

Observation of indirect signals from stable dark matter in the very heavy mass range above 100 TeV requires strongly enhanced annihilation cross sections. Soliton dark matter or bound states can provide a solution to this problem. However, if the field (here a dark isotriplet $\varvec{w}$) which carries the solitons is stable, small particle annihilation cross sections $\sigma _{w\overline{w}}\sim m_w^{-2}$ will create a dark mediator bottleneck which can prevent an observable indirect dark matter signal. This problem can be avoided if the dark mediator $\varvec{w}$ is unstable, e.g. through the $\nu ^2$ portal (21). The neutrino signal for Skyrmion dark matter through the $\nu ^2$ portal could contribute at a subdominant, but potentially noticeable level to the flux of very high-energy neutrinos.

## Footnotes

1 The flux is usually written with the thermally averaged velocity weighted annihilation cross section $\langle \sigma v\rangle (T)$, see also Eq. (1), but heavy dark matter at low redshift is practically at zero temperature. Furthermore, a generic expression $\mathrm{d}\mathcal {N}(E,2M)/\mathrm{d}E$ has been chosen for the fragmentation of an annihilation event with energy $\sqrt{s}=2M$ instead of the customary $2\mathrm{d}\mathcal {N}(E,M)/\mathrm{d}E$, which assumes fragmentation of two initial jets of energy *M*. The reason is that annihilation of bound states or solitons might not necessarily produce only two jets.
